# The queen bee phenomenon in Canadian surgical subspecialties: An evaluation of gender biases in the resident training environment

**DOI:** 10.1371/journal.pone.0297893

**Published:** 2024-03-06

**Authors:** Lydia Goff, Helena Greene, Alexandra Munn, Andrew Furey, Nicholas Smith

**Affiliations:** Division of Orthopedic Surgery, Memorial University, St. John’s, Newfoundland and Labrador, Canada; University of South Florida, UNITED STATES

## Abstract

**Background:**

The queen bee phenomenon (QBP) describes the behavioural response that occurs when women achieve success in a male-dominated environment, and in this position of authority, treat their female subordinates more critically. It has been demonstrated in business, academia, the military, and police force. The goal of this study was to determine whether the QBP occurs in surgical specialties. We hypothesized that female surgeons, fellows, and senior surgical residents would be more critical in their assessment of junior female residents than their male counterparts.

**Methods:**

A scenario-based survey was distributed via email to all Canadian surgical programs between February and March 2021. Scenarios were designed to assess either female or male learners. Centers distributed surveys to attending surgeons, surgical fellows, resident physicians, and affiliate surgeons. Respondents average Likert score for female-based and male-based questions were calculated. Subgroup analyses were performed based on gender, age, seniority, and surgical specialty.

**Results:**

716 survey responses were collected, with 387 respondents identifying as male (54%) and 321 identifying as female (45%). 385 attending surgeons (54%), 66 fellows (9%), and 263 residents (37%) responded. The mean Likert scores for female respondents assessing female learners was significantly lower than male learners (p = 0·008, CI = 95%). During subgroup analysis, some specialties demonstrated significant scoring differences.

**Discussion:**

The QBP was shown to be present among surgical specialties. Female respondents assessed female learners more critically than their male counterparts.

**Conclusion:**

These findings highlight the importance of tackling organizational biases to create more equitable educational and work environment in surgery.

## Introduction

The queen bee phenomenon (QBP) describes the behavioural response that occurs when women achieve success in a male-dominated environment, and in this position of authority, treat their female subordinates more critically. First described in 1973 by Staines et al. [1] regarding women in leadership positions, the QBP has since been shown to exist within academia, business, police force, and military [2–5]. This phenomenon suggests that women who have achieved success in highly hierarchical and gender discriminative workplace environments tend to perpetuate this environment rather than challenge it.

Recent literature suggests that women in positions of power tend to demonstrate more “masculine” qualities associated with leadership, power, success, and professionalism. Ellemers [6] and colleagues indicated that women in academia describe themselves as having as many, if not more, masculine traits than their male colleagues. Further, women in positions of power are more critical of the leadership skills and career commitment of their female subordinates when compared to male subordinates [6,7]. Other research suggests that a self-perpetuating cycle ensues with women in leadership roles reproducing environments that support gender biases [2,8,9].

This phenomenon has been evidenced to be a product of gender disparities and not a result of female traits [10]. Multiple studies, conducted in traditionally male-dominated and hierarchal environments (e.g., academia, military, business, and police forces) confirm its presence [4–7]. To date, no study has quantitatively investigated the role of the QBP in medicine or surgical disciplines. Surgeons and those in surgical training are an understudied and difficult to assess population in psychological research. Learning about the potential existence of the QBP in this discipline can provide much needed insight into the profession’s existing biases, and their role in the training and assessment during training of female learners. This also impacts the career and advancement of women in surgery.

Modern medicine, and specifically surgical disciplines, have been notoriously male-dominated. However, in the last decade, the number of women entering medicine and surgical specialities has grown exponentially [11]. In 2019, Canadian national residency match (CARMS) data [12,13] reflected that an equal ratio of genders entered surgical subspecialties in Canada. Despite this, the Canadian Medical Association [14] indicates that a majority of practicing surgeons across surgical specialties remain men.

The primary goal of this study is to determine if the QBP occurs in surgical subspecialties, as in other professions. We hypothesize that female physicians, in comparison to their male counterparts, will be more critical of female learners, and thus we aim to test whether this phenomenon exists in an applied medical setting or not. Additionally, if the QBP is present, we aim to determine whether its presence is consistent across surgical subspecialties.

## Methods

This was a survey-based study. The survey was granted ethical approval by the Newfoundland and Labrador provincial Health Research Ethics Board (HREB# 2021.0173). Written informed consent was obtained from all study participants prior to completion. Survey participation was voluntary and anonymous. The orthopedic surgery research program at Memorial University provided the funding to purchase two fifty-dollar gift cards. Upon study completion, two study participants were randomly selected to receive the gift cards.

No validated surveys for assessment of the QBP exists in the literature. Based on previous social psychology literature [15–19], question scenarios were designed to explore common workplace interactions between trainees and staff (for example, patient management on the ward, emergency room assessment and management, and resident education). Twenty-five paired scenario-based questions were created. Each scenario was paired with a second matching scenario with the only difference being the learner’s gender pronouns. Half the scenarios were positive interactions and half were negative interactions. A five-point Likert scale was employed to rank respondents’ assessment of each scenario-based interaction with a learner (1 extremely unlikely, 2 somewhat unlikely, 3 neither likely or unlikely, 4 somewhat likely, 5 extremely likely.).

An associate Psychology professor from Memorial University (M.D.), with a research focus in societal cognition, reviewed survey content. Questions were designed to incorporate distractors that would draw attention from the pronoun substitutions and mask the true purpose of the study. Distractors included location of training, personality traits, and personal characteristics (eye and hair colour).

Examples of question scenarios and the complete survey can be found in supplementary data.

The survey was managed through Qualtrics survey software and access was granted to the three primary investigators. Fifty questions were uploaded and stored in a question bank. The larger question bank was created to reduce reliability bias. Individual surveys were designed to take 10–15 minutes to complete to optimize number of responses. The questions were randomized twice to reduce reliability bias. Two blocks of questions were created separating the paired negative and positive scenarios. Each block contained an approximately equal amount of male and female-centered scenarios. Each participant was randomly assigned 13 of 25 questions from each question block, for a total of 26 survey questions in each survey. Question order was randomized for each respondent.

Each survey began with a statement of informed consent, which was required in order to proceed to the questions. The survey included demographic questions to identify level of training (residency, fellowship, or years in practice), age, and gender. Respondents were asked to identify the university of their medical school training, graduation year, residency training institution, and current province of employment. Prior to distribution to the target population, pilot surveys were distributed to all staff and residents in the departments of Anesthesia and Internal Medicine at Memorial University. Participants completed the survey and a debriefing questionnaire. The purpose of the validation trial was to generate feedback and determine whether partial disclosure of the survey was sufficient to conceal the true goal of the study. No trial respondents correctly identified the purpose of the study, and thus no adjustments were made to the survey.

Between February and March 2021, the survey was emailed to all 17 Canadian academic centers with surgical residency programs and was also sent to the national surgical associations. All Canadian surgical specialties were included. Centers were asked to distribute the surveys to attending staff surgeons, surgical fellows, resident physicians, and affiliate surgeons. The survey was closed to responses after June 30, 2021.

Data was exported from Qualtrics into Microsoft Excel. Response data was shared with a research team at the Newfoundland and Labrador Center for Health Information (NLCHI). A statistician employed by NLCHI (P.P.) performed the statistical analysis. Statistical analyses were performed using SPSS and SAS. Descriptive statistics included: means, standard deviations (SDs), ranges, and proportions. The level of significance (α) was set to 0.05, confidence interval (CI) was set at 95%.

Data was divided into a two-by-two matrix of female and male respondents scores of female and male learner interactions.

Post-hoc analyses were performed to assess for differences based on level of seniority, age, and surgical specialty using one-way ANOVA testing. Tukey’s post-hoc test was used to adjust t-values needed based on numbers of comparisons ran given these analyses are subject to elevated family-wise error rates.

A post-hoc power analysis was performed assuming alpha = 0.05, power = 0.8, and an expected effect size of 0.3 (range 0.1–0.5). Given there was no prior similar study examining the QBP, the effect size was conservatively estimated based on several studies examining gender differences in language use [20–22]. Based on the power analysis, at least 352 participants in total (176 of each gender) were needed. This can be compared to the calculated power of the study based on the effect size of 0.218 (within the above reference range) and the large sample size (716 total). The probability of getting a significant result with a one-tailed test was 84.7% (power = 0.847). Therefore, despite the smaller effect size found in the study, the study was appropriately powered to detect differences between male and female assessments.

## Results

### Descriptive statistics

A total of 716 survey responses were collected from staff surgeons, surgical fellows, and residents. Of the 716 respondents, 387 self-reported as male (54.05%), 321 as female (44.83%), one as non-binary (0.14%), and one as “other” (0.14%; Table 1). Six participants did not specify their gender (0.84%). For the purposes of this study, only male and female identifying respondents were included in the analysis. 385 respondents self-reported as staff surgeons (53.77%), 66 as fellows (9.22%), and 263 as residents (36.73%; Table 1). Two survey respondents’ ranking position was listed as “other” and was not specified. The distribution among surgical subspecialties can be found in Table 1.

**Table 1:** Distribution of responders according to gender, surgical position, and specialty.

		No. responses	% total responses
**Gender**	Male	387	54·0
	Female	321	48·83
	Would rather not say	6	0·84
	Non-binary	1	0·14
	Other	1	0·14
**Position**	Staff Surgeon	385	53·77
	Resident	263	36·73
	Fellow	66	9·22
	Other	2	0·28
**Specialty**	Orthopedic Surgery	217	30·31
	Plastic Surgery	101	14·11
	General Surgery	87	12·15
	Obstetrics & Gynaecology	77	10·75
	Ophthalmology	71	9·92
	Urology	54	7·54
	Otolaryngology	28	3·91
	Cardiac Surgery	25	3·49
	Neurosurgery	20	2·79
	Vascular Surgery	17	2·37
	Pediatric Surgery	7	0·98
	Thoracic Surgery	2	0·98
	Other	10	1·40

### Main analyses

A normal distribution of Likert scores was observed for female and male respondents scoring of both female and male-based question scenarios. Of the female respondents, 301 responses were observed for female-centered questions and 299 for male-centered questions. Male respondents answered 361 female-centered questions and 358 male-centered questions. Surveys where respondents did not specify either “male” or “female” as their gender were excluded from analysis for the purposes of this study.

The difference between female respondent scoring of male-centered question scenarios, *M* = 3.328 (*SD =* 0.535), and scoring of female-centered scenarios, *M =* 3.207 (*SD =* 0.573), with equal variances assumed, was shown to be significant *t*(598) *=* 2.664, *p =* .008. Female respondents therefore scored female learners in the female-centered questions more critically than male learners, with a mean difference *M =* -0.121, 95% CI [0.032, 0.210], Cohen’s *d* = 0.218. Male respondents similarly scored female-based scenarios more negatively, but the mean difference was considerably smaller, *M* = -0.024, 95% CI [-0.055, 0.103], and not statistically significant.

One-way ANOVA test was used to assess demographic group differences. Tests were performed using independent variables as the categorical group in question (level of training, age, and subspecialty), with dependent variables being mean scores for all scenarios or differences between mean scores for female and male-based scenarios. Subsequent Tukey’s post-hoc testing was performed to adjust t-values. Mean scores for male-based questions were subtracted from mean scores of female-based questions to obtain a “difference between genders” mean for both female and male respondents. A negative value for a “difference between genders” indicates that male-based scenarios were scored more favorably than female-based scenarios. Resident groups scored learners less critically in all question scenarios than staff surgeons or fellows (Table 2). Resident females scored female learners significantly less favourably than the staff and fellow group (p = 0.036, mean score = -0.110).

**Table 2:** Mean responses in sub categorical One-way Anova analysis: Surgical position.

Position	Respondent gender	No. responses	Mean	Std Dev	P value
Attending Surgeon	Female	132	-0·109	0·640	0·063
	Male	251	-0·021	0·653	0·626
Fellow	Female	36	-0·072	0·639	0·536
	Male	31	0·004	0·661	0·976
Resident	Female	159	-0·110	0·622	0·037
	Male	106	-0·061	0·675	0·394
Other	Female	1	-	-	-
	Male	2	-0·046	0·348	0·883

Both male and female residents, independent of level, scored scenarios more negatively towards female learners, although this did not reach statistical significance.

Results varied throughout subspecialty. Male evaluators in general surgery scored questions with female learners significantly more favorably than male-based scenarios (p = 0.035). Male assessors in ophthalmology scored female scenarios less favourably than male scenarios at a statistically significant level (p = 0.002). Statistically significant findings were discovered in the female orthopedic surgery cohort who scored female learners more critically than male learners (p = 0.049). Both males and female evaluators in neurosurgery scored female learners less positively than their male counterparts. Mean scores from women in neurosurgery were more negative to female learners than men in neurosurgery, however this was not statistically significant (mean female score = -0.450; mean male score = - 0.0094) (Table 3). Obstetrics and gynecology is the only female-dominated specialty based on responses. Mean scores from women in this specialty were more negative to female learners (mean female score = 0.142), however this is not statistically significant. It should be noted that our study is not powered for a subgroup analysis, therefore we may not have found all the relationships that exist because of the smaller group sizes.

**Table 3:** Mean responses in sub categorical One-way Anova analysis: Specialty.

Specialty	Respondent gender	No. responses	Mean	Std Dev	P value
Cardiac Surgery	Female	7	0·092	0·572	0·09
	Male	18	-0·195	0·859	0·348
General Surgery	Female	43	0·061	0·583	0·524
	Male	43	0·205	0·568	0·034
Neurosurgery	Female	2	-0·450	0·636	0·500
	Male	18	-0·009	0·570	0·948
Obstetrics and Gynecology	Female	70	-0·142	0·650	0·078
	Male	5	-0·245	0·724	0·492
Ophthalmology	Female	26	-0·213	0·708	0·137
	Male	43	-0·323	0·576	0·002
Orthopedic Surgery	Female	78	-0·139	0·596	0·049
	Male	139	-0·065	0·683	0·283
Otolaryngology	Female	12	-0·276	0·617	0·150
	Male	16	0·007	0·480	0·955
Pediatric Surgery	Female	6	-0·130	0·825	0·743
	Male	1	-0·293	-	-
Plastic Surgery	Female	48	-0·148	0·736	0·295
	Male	53	0·021	0·635	0·814
Thoracic Surgery	Female	1	-0·343	-	-
	Male	1	-0·400	-	-
Urology	Female	18	0·102	0·479	0·422
	Male	36	0·139	0·735	0·292
Vascular Surgery	Female	6	0·217	0·236	0·075
	Male	10	0.245	0·398	0·101
Other	Female	5	-0·291	0·552	0·303
	Male	4	0·011	0·272	0·943

### Level of evidence

Level V.

## Discussion

The queen bee phenomenon suggests that women who have achieved success in highly hierarchical and gender discriminative workplace environments tend to perpetuate this environment rather than challenge it. As a result, these women are more likely to distance themselves from their female subordinates, treat them more critically, and provide less opportunities and assistance. This behaviour typically functions to elevate the hierarchically-higher woman and hinders the advancement of her subordinate women [1,2,4,8]. To our knowledge no study has quantitatively explored the QBP in medicine. Recognition of the QBP in surgery provides insight into existing biases and the role they play in surgical training and careers. It can allow for the development of strategies to implement change to foster a more equitable training and work environment for female and male surgeons.

Our study identified gender biases within various Canadian academic surgical programs. Female respondents scored female learners (women in a subordinate position in the question scenarios) more critically than male learners. This difference in gender treatment of learners was not found in male respondents. Rather than general gender inequality, these findings suggest the QBP is present in surgical specialties in Canada.

Pervasive societal stereotypes that view women as homemakers and less committed to work are well described in QBP and gender imbalance literature, including in medicine [6,11,23]. It has been postulated that women in positions of power overcome this stereotype by embracing masculine traits [2,7]. Unknowingly, these women perpetuate the QBP by criticizing women as unwilling to undergo similar sacrifices [19]. The princess bee phenomenon described by Kremer at al. [24], highlights this cycle. Junior women do not recognize the behaviours of their senior colleagues and recreate this behaviour when they enter positions of power. When these adverse behaviours are recognized however, the junior woman will often distance herself from female leaders and be less inclined to assume leadership roles. This perpetuates gender-based inequities in leadership roles. Therefore, women achieving success in highly hierarchical and gender discriminative work environments perpetuate this, rather than challenge it [2,25].

Salles and Choo [10] suggest the QBP is a by-product of a gender-biased environment, rather than a result of female traits themselves. Gender-biases are often observed in the medical workplace, specifically in surgical fields [11]. Taken together, this suggests that in medical practice and residency training programs, more favourable assessments would be provided to men than women. The current study found potential evidence of this when women assessed female subordinates.

To our knowledge, this is the first study to demonstrate the presence of the QBP in the medical literature. In the evolving surgical environment, in which women are becoming a greater proportion of the workforce, identification of a gender bias has implications in the assessment of women during training. Evaluations from leadership are central to progression through residency programs, fellowships, transition to practice, and, subsequently, professional success. Without efforts to recognize these biases, they are likely to perpetuate in future training programs. This has shown to be true in academia where an investigation of doctoral candidates and faculty members show there has been no change in the queen bee phenomenon across two studies carried out 15 years apart [6,25]. The current study highlights the existence of systemic organizational gender biases within the understudied medical field and better understanding of their existence should catalyze efforts to eliminate these phenomena.

This study has some limitations. Our survey was developed without pre-existing literature validating survey questionnaires. We attempted to control for this by utilizing experts in the fields of social psychology and by completing validation tests. As with all survey data, there is the potential for respondent bias as well. The survey was voluntary and may not have been representative of all surgical subspecialties. Despite a strong number of responses (n = 716) and an adequately powered study (84.6%), one could postulate if areas of subgroup analysis would have reached statistical significance with a higher response population. Unfortunately given the method of survey distribution via academic surgical residency programs and national surgical associations, we do not know the number of people this survey reached and therefore are unable to calculate a response rate. Generalizability of our results may be limited by an overrepresentation of orthopaedic surgery survey responses (30.3%). This may be due to a better distribution within the orthopaedic surgery community through orthopaedic society newsletters. However, due to the overall large number of responses across specialties, we do not think that this significantly changes our results.

Finally, our study population was restricted to surgical specialties in Canada. To explore these issues more thoroughly, repeat studies in international contexts should be conducted.

## Conclusion

This is the first study to investigate the role of the queen bee phenomenon in surgical specialties. The results demonstrate female respondents treated female learners more critically than their male counterparts. Our findings identify the hidden role that bias has within surgical training. With the knowledge obtained from this study, surgical educators are positioned to implement change and gender equity for future generations of female and male surgeons.

## Supporting information

S1 File Supplemental data: Sample survey questions. (DOCX)

S2 File (PDF)
