# Supplementary material for: The queen bee phenomenon in Canadian surgical subspecialties: An evaluation of gender biases in the resident training environment
Source: PLoS One. 2024 Mar 6;19(3):e0297893. doi: 10.1371/journal.pone.0297893 (PMC10917252; doi:10.1371/journal.pone.0297893)
Supplement: S1 File — (DOCX) [file pone.0297893.s001.docx]

**Supplemental data: Sample survey questions**

**Patient orders on the floor**

1) You note that a 26 year old elective junior resident visiting from Calgary has been

present on the ward doing orders and he is working hard. Attending surgeons seem to

be pleased with his efforts. However, you feel he is not performing at the level you

expect him to be at. You decide to give him more time to improve before you discuss this

with him. (+)

2) You are working with a 28 year old elective junior resident visiting from Edmonton. You

have never worked with her before. However you feel she is not performing at the

expected level for her stage. You are busy during the day attending to cases in the OR.

You decide to still trust that she will notify you of any concerns she has, even without

your supervision. (+)

**Managing ICU patients**

3) You task a vascular surgery resident with rounding on the surgical ICU patients after he was eager for more responsibility. You learn that he hasn’t always been consistent with his plans but overall he has been improving. You decide to critique him about it anyways. (-)

4) You are working with only one other cardiac surgery resident on your team and she has

not always been consistent with her plans but she is improving. There is a call from ICU

to come and reassess a surgical wound of one of your patients that may require

debridement. You do not trust that she is able to manage this on her own. (-)

**Working in an interdisciplinary team (nursing staff)**

5) A 35 year old blond haired junior resident has joined your surgical service. You heard

that he had gotten along well with his co-workers on previous rotations. The OR nurses

let you know that they have some concerns about their interactions with him. You take

note of the concerns, but choose not to discuss them with him at this point. (+)

6) A 29 year old blonde junior resident has just started her first rotation on service. She is

getting along very well with the other residents and attending staff. However some of the

nurses and other healthcare staff (physiotherapists, occupational therapists, social

workers, etc.) on the ward have noted some concerns with their interactions with her.

You keep these concerns in mind, but choose not to bring it up with her at this point. (+)

**Wound checks**

7) You have assigned an enthusiastic and outgoing resident on your service to do one of

the daily wound checks for a patient with a recent amputation and wound healing

concerns. He says the appearance of the wound is now improving. You trust his

judgement rather than re-examining the wound with him. (+)

8) You task an upbeat and energetic resident with completing a wound check for a chronic

patient that you know well. Despite some healing concerns recently, she says the wound

is improving. You decide to trust her judgement and do not find it necessary to

reexamine the wound with her. (+)
