# Supplementary material for: The queen bee phenomenon in Canadian surgical subspecialties: An evaluation of gender biases in the resident training environment
Source: PLoS One. 2024 Mar 6;19(3):e0297893. doi: 10.1371/journal.pone.0297893 (PMC10917252; doi:10.1371/journal.pone.0297893)
Supplement: S2 File — (PDF) [file pone.0297893.s002.pdf]

Demographic questions:

1. Your category:

   Resident

   Attending surgeon

   Fellow

2. Your age group:

   18-24 yo

   25-34 yo

   35-44 yo

   45-54 yo

   55-64 yo

   65 yo +

3. Please select your gender:

   Male

   Female

   Non-binary

4. How much do you identify with your stated gender:

   Not at all

   Slightly

   Somewhat

   Very Strongly

5. If you are a resident:

PGY 1

PYG 2

PGY 3

PGY 4

PGY 5

PGY 6

PGY 7

PGY8+

6. If you are a fellow, please indicate how many fellowships you have completed:

1

2

3

>3

N/A

7. If you are an attending surgeon, years of practice:

0-5 years

5-10 years

11-15 years

16-20 years

21-25 years

> 25 years

N/A

8. Academic institution for medical school:

University of British Columbia

University of Alberta

University of Calgary
University of Saskatchewan

University of Manitoba

Northern Ontario School of Medicine

McMaster University

Queen’s University

University of Western Ontario

University of Ottawa

University of Toronto

Universite Laval

McGill University

Universite de Montreal

Universite de Sherbrooke

Dalhousie University

Memorial University

Outside Canada and the United States, specify: ______

11. Medical school graduation year: ________

12. Academic institution for residency:

University of British Columbia

University of Alberta

University of Calgary

University of Saskatchewan

University of Manitoba

Northern Ontario School of Medicine

McMaster University

Queen’s University

University of Western Ontario

University of Ottawa

University of Toronto

Universite Laval

McGill University

Universite de Montreal

Universite de Sherbrooke

Dalhousie University

Memorial University

Outside Canada and the United States, specify: ______

13. Residency program or field of practice:

Orthopedic surgery

General surgery

Obstetrics and Gynecology

Cardiac Surgery

Vascular Surgery

Neurosurgery

Otolaryngology/ENT (head and neck surgery)

Thoracic Surgery

Pediatric Surgery

Opthalmology

Plastic Surgery

Urology

Anesthesiology

14. Province of current work:

British Columbia

Alberta

Saskatchewan

Manitoba

Ontario

Quebec

New Brunswick

Nova Scotia

Prince Edward Island

Newfoundland and Labrador

Yukon

Northwest Territories

Nunavut

15. If you are a resident, average number of residents you work with in a week

0-5

6-10

11-15

16-20

20 +

N/A

16. If you are a fellow: average number of residents you work with in a week

0-5

6-10

11-15

16-20

20 +,

N/A

17. If you are an attending surgeon: average number of residents you work with in a week

0-5

6-10

11-15

16-20

20 +

N/A

Patient orders on the floor

1) You note that a 26 year old elective junior resident visiting from Calgary has been present on the ward doing orders and he is working hard. Attending surgeons seem to be pleased with his efforts. However, you feel he is not performing at the level you expect him to be at. You decide to give him more time to improve before you discuss this with him.

Almost always true	Usually True	Occasionally	Usually not true	Almost never true
1	2	3	4	5

2) You are working with a 28 year old elective junior resident visiting from Edmonton. You have never worked with her before. However you feel she is not performing at the expected level for her stage. You are busy during the day attending to cases in the OR. You decide to still trust that she will notify you of any concerns she has, even without your supervision.

Almost always true	Usually True	Occasionally	Usually not true	Almost never true
1	2	3	4	5

Managing ICU patients

3) You task a vascular surgery resident with rounding on the surgical ICU patients after he was eager for more responsibility. You learn that

he hasn't always been consistent with his plans but overall he has been improving. You decide to critique him about it anyways.

Almost always true	Usually True	Occasionally	Usually not true	Almost never true
1	2	3	4	5

4) You are working with only one other cardiac surgery resident on your team and she has not always been consistent with her plans but she is improving. There is a call from ICU to come and reassess a surgical wound of one of your patients that may require debridement. You do not trust that she is able to manage this on her own.

Almost always true	Usually True	Occasionally	Usually not true	Almost never true
1	2	3	4	5

Presentations

5) You assign a resident trained in British Columbia to present a case at interdisciplinary rounds on behalf of the surgical team. He gave a reasonable presentation, however he left out several details about the long term plan. You decide to critique him about those particular elements of his presentation.

Almost always true	Usually True	Occasionally	Usually not true	Almost never true
1	2	3	4	5

6) One of the junior residents who was trained in Manitoba is presenting a case at a research conference on your behalf. She has worked hard and gave a reasonable presentation, however her summary and conclusions should have been clearer. You decide that it's important to point that out to her.

Almost always true	Usually True	Occasionally	Usually not true	Almost never true
1	2	3	4	5

Working in an interdisciplinary team (nursing staff)

7) A 35 year old blond haired junior resident has joined your surgical service. You heard that he had gotten along well with his co-workers on previous rotations. The OR nurses let you know that they have some concerns about their interactions with him. You take note of the concerns, but choose not to discuss them with him at this point.

Almost always true	Usually True	Occasionally	Usually not true	Almost never true
1	2	3	4	5

8) A 29 year old blonde junior resident has just started her first rotation on service. She is getting along very well with the other residents and attending staff. However some of the nurses and other healthcare staff (physiotherapists, occupational therapists, social workers, etc.) on the ward have noted some concerns with their interactions with her. You keep these concerns in mind, but choose not to bring it up with her at this point.

Almost always true	Usually True	Occasionally	Usually not true	Almost never true
1	2	3	4	5

Seeing clinic follow ups

9) You are running a busy emergency clinic over the holidays. The clinic is full and you and your only resident - who is away from home for the holidays - are both struggling to keep on schedule and review all cases. He has demonstrated reasonable knowledge, management and plans in the past. You entrust him with seeing some of the easier follow ups alone without reviewing them with him.

Almost always true	Usually True	Occasionally	Usually not true	Almost never true
1	2	3	4	5

10) You are in a clinic with a resident with whom you have worked before - they moved from their hometown for residency. There is a minor procedure to be done for one of the patients. You have observed her perform this procedure only once before, but she performed reasonably well at that time. The clinic is quite busy. You are happy to entrust her with this procedure alone and do not feel the need to supervise her.

Almost always true	Usually True	Occasionally	Usually not true	Almost never true
1	2	3	4	5

Pre-op assessment

11) You assign a locally-trained co-resident to do a pre-operative assessment for a patient for the OR today. He does a good assessment but misses a key part of the history. You know that he was on call overnight and barely slept and he is working hard to perform well whilst exhausted. You feel that it is important to stress his errors as a teaching point.

Almost always true	Usually True	Occasionally	Usually not true	Almost never true
1	2	3	4	5

12) One of the locally-trained junior residents is seeing a few of your cases in the pre-operative clinic. Overall, she has done a good job, but on a few patients she has missed some important parts of the assessment. You know she has had a busy week and is tired. You feel that it is necessary to point out her omissions for teaching purposes.

Almost always true	Usually True	Occasionally	Usually not true	Almost never true
1	2	3	4	5

Pre-op change in clinical status

13) An in-patient is awaiting surgery. They have been fully cleared for the OR and are scheduled for the first case in the morning. At 2 am you receive a call from your visiting international resident, and he is concerned about the patient. They are stable, however he has some concerns about a perceived change in the patient's status. He would like them reassessed. You saw the patient a few hours ago and they were stable. You disregard his concerns, and decide to proceed with surgery.

Almost always true	Usually True	Occasionally	Usually not true	Almost never true
1	2	3	4	5

14) You saw a patient in the ED earlier. They were admitted and fully cleared for the OR tomorrow. Late that night, you receive a phone call from the visiting international resident on your service. She is very anxious about a perceived change in clinical status and she wants you to reassess the patient and plan. Based on her description, the patient is stable. You disregard her concerns, and decide to proceed with surgery as planned in the morning.

Almost always true	Usually True	Occasionally	Usually not true	Almost never true
1	2	3	4	5

Wound checks

15) You have assigned an enthusiastic and outgoing resident on your service to do one of the daily wound checks for a patient with a recent amputation and wound healing concerns. He says the appearance of the wound is now improving. You trust his judgement rather than re-examining the wound with him.

Almost always true	Usually True	Occasionally	Usually not true	Almost never true
1	2	3	4	5

16) You task an upbeat and energetic resident with completing a wound check for a chronic patient that you know well. Despite some healing concerns recently, she says the wound is improving. You decide to trust her judgement and do not find it necessary to reexamine the wound with her.

Almost always true	Usually True	Occasionally	Usually not true	Almost never true
1	2	3	4	5

Dressing changes

17) You have instructed a shy and introverted resident to change the dressing of a patient with a recent revision surgery for infection. He confidently says it looks healthy. Yesterday you weren't pleased with the wound appearance, but he tells you it looks much better today. You decide to reexamine the wound today rather than accept his assessment.

Almost always true	Usually True	Occasionally	Usually not true	Almost never true
1	2	3	4	5

18) Your resident, who is fairly meek and reserved, is examining a patient who has had ongoing wound management issues. Based on her assessment, she thinks the wound appears healthy and suggests a particular dressing change. Rather than accepting her suggestions, you reexamine the wound yourself.

Almost always true	Usually True	Occasionally	Usually not true	Almost never true
1	2	3	4	5

Managing a trauma team

19) You are observing a keen and confident resident running one of his first trauma codes. He was assertive and was able to keep control of the code. The code was reasonably well managed but should have been run more efficiently. You decide it's important to highlight his errors as a teaching point.

Almost always true	Usually True	Occasionally	Usually not true	Almost never true
1	2	3	4	5

20) You observe an assertive and bossy junior resident running a trauma code. She has not had much experience as Trauma Team Lead. She performs reasonably well. She does not omit anything that would be life threatening to the patient, however she hesitated through a few of the steps. You decide it's important to highlight her hesitations as you feel it is a good teaching point.

Almost always true	Usually True	Occasionally	Usually not true	Almost never true
1	2	3	4	5

21) The over-confident urology resident on the trauma service has now seen you run several trauma codes. He would like to serve as Trauma Team Lead for the next code. Despite his junior status, you are happy to let him serve this role.

Almost always true	Usually True	Occasionally	Usually not true	Almost never true
1	2	3	4	5

22) Your self-assured general surgery junior resident notifies you of an impending trauma code. She has seen you run a few codes so far, but has not run any alone. Despite being a junior resident, you are comfortable with letting her lead the code.

Almost always true	Usually True	Occasionally	Usually not true	Almost never true
1	2	3	4	5

Emergency consultation and evaluating the need for surgical intervention

23) You are on call with a 34 year old orthopedic resident this evening.

He has seen and assessed an emergency consultation, obtained operative consent, and presented the patient to you. The OR is available now. You trust his judgement and plan to take the patient to the OR and do a brief assessment in holding.

Almost always true	Usually True	Occasionally	Usually not true	Almost never true
1	2	3	4	5

24) You hear from your 30 year old plastic surgery resident in the middle of the night. They review a consultation with you. She reviews the case with you and based on her impressions, she wants to bring the patient emergently to the OR. You choose to heed your resident's impressions for now and head in to the hospital to assess the patient in OR holding.

Almost always true	Usually True	Occasionally	Usually not true	Almost never true
1	2	3	4	5

New patient clinic referrals

25) The brown-haired OBGYN resident that you are working with has seen a new patient who is keen to proceed with surgery. He gives you a synopsis of his consultation. He has proven to be a reliable resident in the past. However, you repeat the history and physical exam, rather than trust his assessment.

Almost always true	Usually True	Occasionally	Usually not true	Almost never true
1	2	3	4	5

26) A brown-haired opthomology resident with whom you have worked with previously is in clinic with you today. In the past she has proved to be reliable. She sees a new patient consultation and proceeds to perform an appropriate minor procedure based on her impressions without reviewing the case with you first. You reprimand her for proceeding without speaking with you.

Almost always true	Usually True	Occasionally	Usually not true	Almost never true
1	2	3	4	5

Assessment of a "sick patient"

27) The Ontario-trained ENT resident you are working with overnight calls you to review a consult. He is worried about the patient needing an emergent operation, though the consult he presents seems like it could be done in the morning. You trust his judgement and come in yourself to see the patient.

Almost always true	Usually True	Occasionally	Usually not true	Almost never true
1	2	3	4	5

28) The Neurosurgery resident from Quebec that you are working with overnight calls you about a patient that has been stable on the floor. She has concerns about the patient deteriorating and would like you to assess them urgently. You trust her judgement and come in to assess the patient.

Almost always true	Usually True	Occasionally	Usually not true	Almost never true
1	2	3	4	5

Patient death

29) An inpatient on your unit suffers a sudden unexpected cardiac event overnight resulting in their death. Your loyal and reliable junior resident ran a prolonged and difficult resuscitation. Your first discussion with him is about whether or not he had missed any impending symptoms and if his patient's death could have therefore been avoided.

Almost always true	Usually True	Occasionally	Usually not true	Almost never true
1	2	3	4	5

30) A new consultation that your dutiful and trustworthy resident is

currently assessing in the ED suffers a sudden episode of severe respiratory distress midway through the assessment. The patient codes. She takes charge and leads a prolonged code ending in the patient's death. The first conversation you have with her is whether she missed anything in her assessment that could have prevented the patient's demise.

Almost always true	Usually True	Occasionally	Usually not true	Almost never true
1	2	3	4	5

31) Your Memorial University-trained junior resident has been caring for an inpatient who has had a lengthy and complex admission. Their status has been deteriorating and it is expected that the patient will not survive. You know that he has had discussions with the family about palliative care, but they have been reluctant. When the patient dies, the course is prolonged unnecessarily. You understand that he did his best to try and palliate the patient and that the obstacles were not his fault.

Almost always true	Usually True	Occasionally	Usually not true	Almost never true
1	2	3	4	5

32) Your Dalhousie-trained resident has been personally taking care of a longstanding inpatient. Their health has been gradually deteriorating and she has had multiple conversations with the family and multidisciplinary team about moving towards palliative care. The family has been reluctant in every conversation with her. When the time comes, the patient's death was unnecessarily drawn out. You realise that she did her best in that context and that the complications that ensued were out of her hands.

Almost always true	Usually True	Occasionally	Usually not true	Almost never true
1	2	3	4	5

WHO peri-operative briefing

33) A 24 year old junior resident new to your service, who happens to be an avid cyclist, is joining you in today's elective OR room. He is confident to do the WHO pre-operative briefing. You let him do this, however he misses the allergy review. You feel it is important to emphasize to him that his mistake could cause patient harm.

Almost always true	Usually True	Occasionally	Usually not true	Almost never true
1	2	3	4	5

34) One of the 32 year old junior residents, who happens to be an avid runner, completes the WHO peri-operative briefing. Overall she does well, however she does not ensure that a key piece of equipment is in the room. You feel that it is important to tell her her mistake could cause patient harm if the procedure was started without the appropriate equipment present.

Almost always true	Usually True	Occasionally	Usually not true	Almost never true
1	2	3	4	5

Operating independently

35) A pediatric surgery resident from Saskatchewan known to you is in your OR. The case is routine and you have guided him through the procedure multiple times before and he performed well. He is keen to operate more independently. You are likely to remain hands off throughout the operation. Once the procedure is almost finished, you are happy to break scrub.

Almost always true	Usually True	Occasionally	Usually not true	Almost never true
1	2	3	4	5

36) One of the pediatric surgery residents from New Brunswick with whom you have operated on multiple occasions is in your OR today. It is a routine procedure that you have seen her perform well several times in the past. You get called out of the OR for an urgent matter. The case is

almost finished and she tells you she is comfortable completing the procedure. You leave the OR and allow your resident to finish the procedure.

Almost always true	Usually True	Occasionally	Usually not true	Almost never true
1	2	3	4	5

Closing the wound independently

37) A blue-eyed resident you have worked with several times was assisting you in the OR today. He was engaged throughout the case. At the end of the procedure, you are happy to leave the room and let him close the wound unsupervised.

Almost always true	Usually True	Occasionally	Usually not true	Almost never true
1	2	3	4	5

38) A brown-eyed resident with whom you have operated before has demonstrated that she sutures well. You feel comfortable to let her close the wound unsupervised at the end of all your cases together.

Almost always true	Usually True	Occasionally	Usually not true	Almost never true
1	2	3	4	5

Managing conflict/complaints

39) A general surgery resident complains to you about one of their co-residents. He says that their scheduling of call duties has been unfair and asks for you to intervene. Other residents have found their call schedules fair in the past. You are inclined to leave him to sort it out with his fellow resident.

Almost always true	Usually True	Occasionally	Usually not true	Almost never true
1	2	3	4	5

40) Some residents have brought to you their concerns about one of their neurosurgery co-residents. They brought up some difficult interactions with her. However, all of the attending staff seem to like her very much and have not noticed any such issues. You decide to leave it be for now, and let her sort it out with her fellow resident.

Almost always true	Usually True	Occasionally	Usually not true	Almost never true
1	2	3	4	5

Resident education

41) You are the lead on the trauma service. A resident, who is also doing a Master's in clinical education, asks you to do a teaching session with his co-residents on ATLS. He says that he will organize the session. You are very busy with your own practice but you agree to help him.

Almost always true	Usually True	Occasionally	Usually not true	Almost never true
1	2	3	4	5

42) You are provided a new academic teaching position. It is an exceptionally busy time for you right now and one of the residents who is also doing their Master's in surgical education asks you to host a seminar. She says she will help organise the session, but it does require your time. You still agree to help her with the presentation.

Almost always true	Usually True	Occasionally	Usually not true	Almost never true
1	2	3	4	5

43) You are operating with a locally-trained vascular surgery resident today and quizzing them throughout the case. It is clear that he isn't at his expected level and has not done his readings in preparation for your case together. You were told by a colleague that he has had some challenging personal events lately. You continue to quiz him since it is a good opportunity for him to learn.

Almost always true	Usually True	Occasionally	Usually not true	Almost never true

1	2	3	4	5

44) You are running teaching rounds and one of the locally-trained orthopedic residents is clearly struggling through her questions. It is apparent that she hasn't done her readings. You remember hearing that she has been struggling with some difficult events in her personal life. You continue to let her struggle through the case, as it is a better opportunity for her to learn.

Almost always true	Usually True	Occasionally	Usually not true	Almost never true
1	2	3	4	5

Assistance with research projects

45) A 31 year old black-haired OBGYN resident approaches you with an idea for his research project. He asks for your assistance with formulating a research question. You are extremely busy with work, however you are keen to help him.

Almost always true	Usually True	Occasionally	Usually not true	Almost never true
1	2	3	4	5

46) A 26 year old blonde-haired ENT resident has been working hard on her research project from the beginning of her residency. She asked you for assistance in the editing process. You are already busy, however you find time to help her.

Almost always true	Usually True	Occasionally	Usually not true	Almost never true
1	2	3	4	5

Supervision of research projects

47) An internationally-trained ophthalmology resident approaches you about supervising his research project. He is quite adamant that he

needs your help to get the project underway. You are very busy with your practice, so you refuse and suggest he attempts to start the project himself first.

Almost always true	Usually True	Occasionally	Usually not true	Almost never true
1	2	3	4	5

---

48) You already have a sufficient amount of research projects underway with other residents. One of the junior internationally-trained urology residents comes to you with an idea for her first project. Her idea is quite a good one but needs a lot of work. You refuse her offer and suggest she develops her project further before approaching staff.

Almost always true	Usually True	Occasionally	Usually not true	Almost never true
1	2	3	4	5

Pre-op anesthesia consults

49) You task a happy-go-lucky, eager cardiac resident with completing a pre-operative assessment for a patient for the OR today. He does a fair job, but should have been more thorough. You observe that he has been distracted lately since he has an upcoming exam. You feel it is important to tell him that his performance is lacking.

Almost always true	Usually True	Occasionally	Usually not true	Almost never true
1	2	3	4	5

50) A friendly, light-hearted plastic surgery resident on your service was assigned a preoperative assessment of a patient you know well. She finishes this and reviews with you. You note she missed a key piece of the surgical history. She has been distracted lately while planning for her electives. You feel she needs to know that your expectations were higher than how she performed today.

Almost always true	Usually True	Occasionally	Usually not true	Almost never true

1	2	3	4	5
